# Control of Pore Sizes in Epoxy Monoliths and Applications as Sheet-Type Adhesives in Combination with Conventional Epoxy and Acrylic Adhesives

**DOI:** 10.3390/molecules29092059

**Published:** 2024-04-29

**Authors:** Yoshiyuki Kamo, Akikazu Matsumoto

**Affiliations:** 1 Advanced Technology R&D Center, Mitsubishi Electric Corporation, 8-1-1, Tsukaguchi-Honmachi, Amagasaki, Hyogo 661-8661, Japan; 2 Department of Applied Chemistry, Graduate School of Engineering, Osaka Prefecture University, 1-1 Gakuen-cho, Naka-ku, Sakai 599-8531, Osaka, Japan; 3 Department of Applied Chemistry, Graduate School of Engineering, Osaka Metropolitan University, 1-1 Gakuen-cho, Naka-ku, Sakai 599-8531, Osaka, Japan

**Keywords:** monolith, epoxy resin, porous structure, mechanical property, solubility parameter, adhesive property

## Abstract

Materials with monolithic structures, such as epoxy monoliths, are used for a variety of applications, such as for column fillers in gas chromatography and HPLC, for separators in lithium-ion batteries, and for precursor polymers for monolith adhesion. In this study, we investigated the fabrication of epoxy monoliths using 1,3-bis(*N,N*-diglycidylaminomethyl)cyclohexane (TETRAD-C) as the tetrafunctional epoxy and 4,4′-methylenebis(cyclohexylamine) (BACM) as the amine curing agent to control pore diameters using polyethylene glycols (PEGs) of differing molecular weights as the porogenic agents. We fabricated an epoxy monolith with micron-order pores and high strength levels, and which is suitable for the precursors of composite materials in cases where smaller PEGs are used. We discussed the effects of the porous structures of monoliths on their physical properties, such as tensile strength, elongation, elastic modulus, and glass transition temperatures. For example, epoxy monoliths prepared in the presence of PEGs exhibited an elastic modulus less than 1 GPa at room temperature and Tg values of 175–187 °C, while the epoxy bulk thermoset produced without any porogenic solvent showed a high elastic modulus as 1.8 GPa, which was maintained at high temperatures, and a high Tg of 223 °C. In addition, the unique adhesion characteristics of epoxy monolith sheets are revealed as a result of the combinations made with commercial epoxy and acrylic adhesives. Epoxy monoliths that are combined with conventional adhesives can function as sheet-type adhesives purposed with avoiding problems when only liquid-type adhesives are used.

## 1. Introduction

A monolith is a highly porous material with a co-continuous structure that consists of three-dimensionally continuous through-holes and a network frame [1,2,3,4]. Materials with a monolithic structure are attracting attention as separation media used in chromatography, immobilized catalysts, adsorbents, and sensors [5,6,7,8,9]. In addition, polymer monoliths can be easily modified by functional groups or the usage of filler additives, although they are inferior to inorganic monoliths in terms of heat resistance and mechanical strength. The development and application of polymer monoliths using epoxy, acrylics, and styrene are thus underway [10,11,12,13].

Generally, organic monoliths are formed through microphase separation processes classified into the categorizations of polymerization-induced phase separation (PIPS) and nonsolvent-induced phase separation. Epoxy monoliths are one of the co-continuous porous materials formed by PIPS, and the phase separation is caused by spinodal decomposition during the heat curing of the epoxy resin and crosslinker in the presence of porogenic solvents [3,14,15,16,17,18,19]. The phase-separated structure of epoxy resin is fixed as a result of crosslinking reactions. Therefore, the rate of the crosslinking reaction greatly influences the shape and pore size of the monolith [20,21]. Efforts are being made to take advantage of continuous pores and to arrange catalysts on monoliths, imitating monoliths so as to increase their activity levels [22,23].

In recent years, epoxy monoliths have been used for the construction of composite materials. Shimizu et al. improved the tribological properties of monolithic polymer films by tuning a fine pore structure for soft and resilient porous polymer lubricant systems [24]. Tominaga et al. reported on the mechanical strength and filling conditions using X-ray CT when a second component was placed inside the porous materials [25,26]. Furthermore, Sugimoto et al. provided a report on a monolith bonding method with an advantage of a continuous porous structure [27,28]. The anchor effect is used to increase adhesive strength by creating an epoxy monolith on the adherend when bonding difficult-to-adhere objects or dissimilar materials. In these applications, monolith pores are filled with liquid or viscous materials, and the incorporated materials are then solidified or cured. Thus, controlling the pore sizes and three-dimensional structures of monoliths is important. It is also necessary to consider increasing the tensile strength of the monolith and improving its heat resistance so that it can be deployed for usage in various environments. A tetrafunctional epoxy was used to increase the crosslinking density and improve upon both the heat resistance and toughness compared to a monolith using a difunctional epoxy [29,30]. Meanwhile, previous reports have shown that monoliths using tetrafunctional compounds have small pore diameters and are not suitable for applications filling any polymer material in the monolith [31,32]. It has already been reported that it is possible to form an appropriate pore size by changing the fabrication conditions of monoliths [19,26,33].

An adhesion process often requires spacers, such as polymer or glass beads, which are used for the controlling of adhesive thickness. In addition, the viscosity or fluidity of adhesives should be finely justified since conventional adhesives are supplied in a liquid form [34]. An epoxy monolith sheet with a unique porous structure is expected to function as the gap adjuster for adhesives by the combination with commercial epoxy and acrylic adhesives. Studies already show that it is possible to form an appropriate pore size by changing the fabrication conditions of monoliths. It would be convenient if the application process could be completed by simply putting things together, such as double-sided pressure-sensitive adhesive (PSA) tape [35,36,37]. Here, we described the results for the controlling of the porous structure and the physical properties of the epoxy monolith for constructing a new kind of sheet-type adhesive resulting from combinations with liquid-type adhesives. It was also expected that a new type of bonding using epoxy and acrylic adhesives in combination with epoxy monolith sheets would be achieved, because there are great operational advantages for using a liquid adhesive as the material impregnated in the epoxy monolith sheet. In the case of the single-use of liquid adhesives, it is actually necessary to regulate the application procedures and carefully control the thickness of the adhesives, but these troublesome and time-consuming problems will be avoided with the new adhesion method using the monolith sheets. The validity of monolith sheets as porous molds deployed for adhesion using epoxy and acrylics as liquid adhesives has thus been demonstrated.

## 2. Experimental Method

### 2.1. Fabrication of Epoxy Monoliths

**Figure 1:**
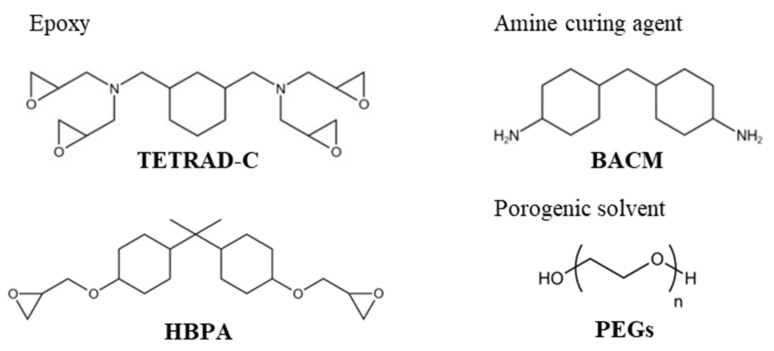
Chemical structures of the materials used in this study.

The chemical structures of the reagents used in this study are shown in Figure 1. 1,3-Bis(*N,N*-diglycidylaminomethyl)cyclohexane (TETRAD-C) manufactured by Mitsubishi Gas Chemical Corporation, Ltd., Tokyo, Japan, was used as the base epoxy resin. Hydrogenated bisphenol-A type epoxy resin (HBPA) manufactured by New Japan Chemical Corporation, Ltd., Osaka, Japan, was used as the additive. 4,4′-Methylenebis(cyclohexyl-amine) (BACM) was purchased from Tokyo Chemical Industry Corporation, Ltd., Tokyo, Japan, and was used as the crosslinking agent. Ethylene glycol (PEG50), diethylene glycol (PEG100), triethylene glycol (PEG150), and polyethylene glycol (PEG, Mn = 200), which were used as the porogenic solvents, were purchased from Tokyo Chemical Industry Corporation, Ltd., Tokyo, Japan. Table 1 shows the solubility parameters of the chemicals [38]. All reagents and solvents were used without further purification, unless stated otherwise. Polyvinyl alcohol (PVA, Mn = 500) was purchased from Fujifilm Wako Pure Chemical Corporation, Ltd., Osaka, Japan, and was used as the sacrificial layer placed on the glass plate for easily peeling off the monolith sheet after the fabrication process.

Figure S1, which is contained within the Supplementary Materials, shows the procedure for preparing the epoxy monolith sheets. The monolith sheet was prepared as follows. A solution of 1 wt% PVA in ion-exchanged water was applied onto the glass and heated at 110 °C for 1 h to create a sacrificial layer of PVA on the glass surface. TETRAD-C, HBPA, BACM, and a porogen (70 wt% for PEGs) were mixed well for 10 min using a planetary centrifugal mixer (ARE-250; Thinky Corporation, Ltd., Tokyo, Japan) at a ratio of 2[NH2]/[epoxy] (γ value) = 1.0. The mixture of the chemicals was spread onto the PVA-coated glass plate and sandwiched with another glass plate while maintaining the desired thickness. The reaction mixture was cured at 130 °C for 30 min using an oven. After curing, the samples were washed with ion-exchanged water using ultrasonics for 15 min to remove the porogenic solvent. They were then stored in water overnight. The samples were dried at 40 °C for 12 h in a vacuum oven (LCV-233; Espec Corporation, Ltd., Osaka, Japan). The thickness of the epoxy monolith was determined using a digital thickness gauge (547–401; Mitutoyo Corporation, Ltd., Kanagawa, Japan). Epoxy monolith sheets with thicknesses of 200–250 μm and 80–120 μm were used for the measurement of physical properties and for the adhesive tests, respectively.

### 2.2. Measurements

The differential scanning calorimetry (DSC) measurements were carried out using a DSC-X7000 (Hitachi High-Tech Corporation, Ltd., Tokyo, Japan) with a heating rate of 10 °C/min to determine the curing temperature in a sealed pan. The epoxy monolith sheet was carefully cut using a sharp blade after cooling it with liquid nitrogen. The cross-section of the epoxy monolith was observed using a TM4000 plus (Hitachi High-Tech Corporation Ltd., Tokyo, Japan) with an acceleration voltage of 15 kV and with Pt vapor deposition for pretreatment. Monolith sheets prepared under different conditions were cut into strips of 10 mm × 40 mm using a stainless-steel cutter. The tensile test was carried out at room temperature with a tensile rate of 1.0 mm/min using an Autograph AGS-10KN X (Shimadzu Corporation, Ltd., Kyoto, JAPAN). The dynamic mechanical analysis (DMA) measurement was carried out using an EXSTER6000 (Hitachi High-Tech Corporation, Ltd., Tokyo, Japan) in an atmospheric environment to examine the glass transition temperatures and storage modulus of the monolith sheets. This test was conducted in a tensile mode, at a heating rate of 2 °C/min and a frequency of 1 Hz.

### 2.3. Adhesion Test

**Figure 2:**
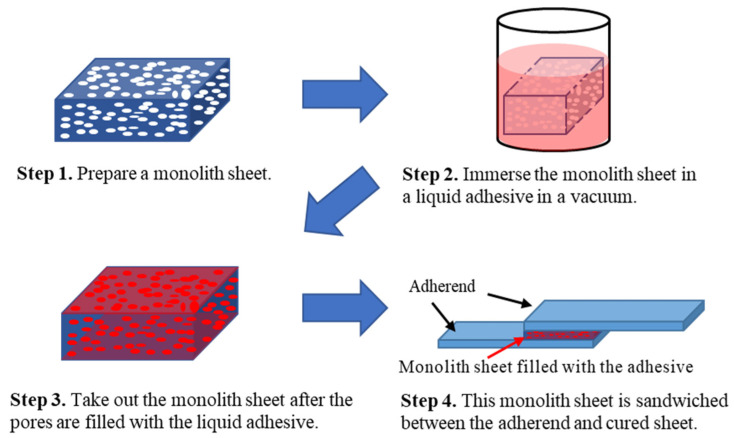
The procedure for a sheet-type adhesive structure using an epoxy monolith sheet in combination with liquid-type conventional adhesives.

Steel Plate Cold Commercial (SPCC) produced by Engineering Test Service Corporation, Ltd., Hyogo, Japan, was used as the adherend for the measurement of the lap-shear adhesion strength. The surface of the SPCC was wiped with acetone and modified with deep ultraviolet light using a low-pressure mercury lamp (11–13 mW/cm^2^) for 5 min before the preparation of the test pieces. The specimens were prepared according to the ISO 4587 method [39]. The adhesive area used was 25.0 mm × 12.5 mm. The adhesive thickness was adjusted at ca. 0.1 mm using glass beads or monolith sheets. Figure 2 shows the procedure for adhesive structures consisting of commercial liquid-type adhesives and the epoxy monolith used as the porous sheet. The commercial adhesives used in this study were either heat curing-type epoxy adhesives or acrylic adhesives. Epoxy adhesive (CLS-1194) manufactured by ADEKA Corporation, Ltd., Tokyo, Japan, was used, and an acrylic adhesive (AS-6704) was purchased from Asec Corporation, Ltd., Kanagawa, Japan. The adhesives were cured under the conditions recommended by each manufacturer. CLS-1194 was cured at 180 °C for 30 min and AS-6704 was cured at 120 °C for 30 min. The tensile test was carried out at room temperature at a rate of 10 mm/min.

## 3. Results and Discussion

### 3.1. DSC Measurement

Table 2 shows the results of the DSC measurement for an epoxy curing system consisting of TETRAD-C, BACM, and PEGs in the absence and presence of HBPA used as an additional epoxy. The onset and peak temperatures and the enthalpy values of the exothermic reactions observed during thermal curing increased as the molecular weight of glycol increased. This was interpreted by the acceleration of epoxy curing in a polar medium or circumstance [40,41,42]. For example, strong hydrogen-bonding compounds (such as alcohols) can stabilize transitions for the curing reactions and accelerate the nucleophilic attack of amines. The acceleration of curing reactions by hydroxy groups has been theoretically and experimentally demonstrated in the literature [43,44,45,46,47,48]. These results support the idea that hydrogen bonding plays a more significant role in assisting the curing reaction between epoxy and amine when glycols with a low molecular weight are used.

As the molecular weight of PEGs becomes lower, the number of hydroxy groups that can come into contact with an epoxy group increases. The reaction gradually started just after the reagents were mixed at room temperature when PEG50 was used. As a result, phase separation occurs more frequently because the SP values of low-molecular-weight PEGs are larger than those of the epoxies and amines, as shown in Table 1. 

On the other hand, the enthalpy value decreased without changing the reaction temperature when HBPA was added. This was because the number of epoxy groups included in the curing system decreased as the HBPA content of the bifunctional epoxy increased. It is likely that the reactivities of the glycidyl groups of TETRAD-C and HBPA are similar to each other, resulting in the constant onset and peak temperatures of the DSC curves.

### 3.2. SEM Observations

**Figure 3:**
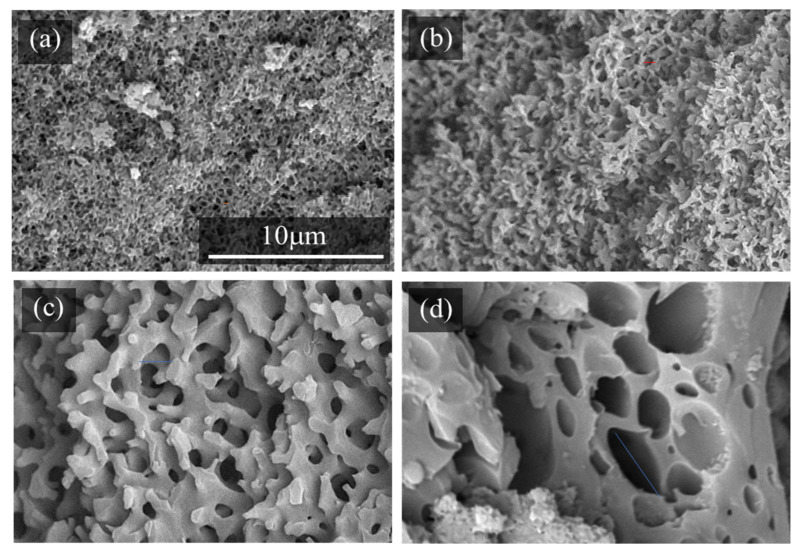
Cross-sectional SEM images of the epoxy monolith fabricated from the TETRAD-C/BACM/PEG system using different PEGs after curing at 130 °C for 30 min: (**a**) PEG200, (**b**) PEG150, (**c**) PEG100, and (**d**) PEG50.

Figure 3 shows the cross-sectional SEM images of the epoxy monoliths, which were fabricated using different PEGs with curing at 130 °C for 30 min. Based on the cross-sectional images, it is clear that the pore diameters increase as the molecular weights of the PEGs decrease. PEGs with lower molecular weights have solubility parameter values that are larger than those for TETRAD-C and BACM, as shown in Table 1. The pore and monolith skeleton sizes change depending on the rate of Ostwald ripening during the phase separation of the epoxy monolith curing system in the presence of PEGs as porogenic solvents [19,33]. When there is a high level of miscibility of the epoxy or the amine in relation to the porogenic solvents, phase separation occurs more slowly and the domain size of the phase-separated layers decreases. The phase-separated structure is fixed at any stage of the curing reaction due to the formation of the crosslinked network structure of the epoxy. When PEG50 is used, the pores are too large and the epoxy monolith is difficult to maintain in the shape of the sheet.

**Figure 4:**
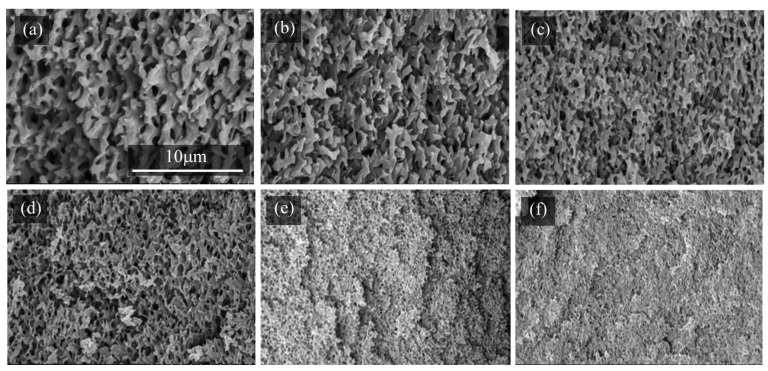
Cross-sectional SEM images of the epoxy monolith fabricated from the TETRAD-C/BACM/PEG200 system at different curing temperatures for 30 min: (**a**) 90 °C, (**b**) 100 °C, (**c**) 110 °C, (**d**) 120 °C, (**e**) 140 °C, and (**f**) 160 °C. See Figure 3a for the image of the monolith obtained at 130 °C.

We also observed the changes in pore sizes depending on the curing temperature. Figure 4 shows the cross-sectional SEM images of the epoxy monolith obtained using PEG200 at various temperatures. The lower the curing temperature, the larger the pores of the monolith. Similar observations of the temperature effects were reported in the literature [24,32,34]. The magnitude of the change occurring as a result of different curing temperatures is moderate when compared with the drastic changes in pore sizes controlled by the molecular weights of the PEGs.

**Figure 5:**
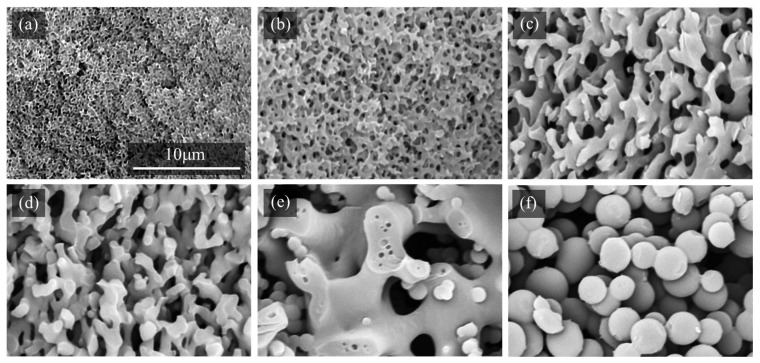
Cross-sectional SEM images of the epoxy monolith fabricated from the TETRAD-C/HBPA/BACM/PEG200 system cured at 130 °C for 30 min: (**a**) HBPA 0 mol% (**b**) 5 mol%, (**c**) 10 mol%, (**d**) 15 mol%, (**e**) 20 mol%, and (**f**) 30 mol%.

Figure 5 shows the additional effect of HBPA on the TETRAD-C/BACM/PEG200 curing system. The pore diameter of the monoliths increased according to the content of HBPA present in the system. When 30 mol% of HBPA was added, aggregates of particles were produced and the crumbly monolith sheet could not stand by itself. In this case, the phase separation mode changed from the spinodal decomposition mechanism to the nucleation and growth mechanism [49,50] when a large amount of HBPA was added. The drastic change in the pore size is accounted for by the retardation of the fixing of the phase-separated structure by the addition of HBPA as the difunctional epoxy to the curing system of TETRAD-C as the tetrafunctional epoxy.

### 3.3. Mechanical and Physical Properties of the Monolith Sheets

**Figure 6:**
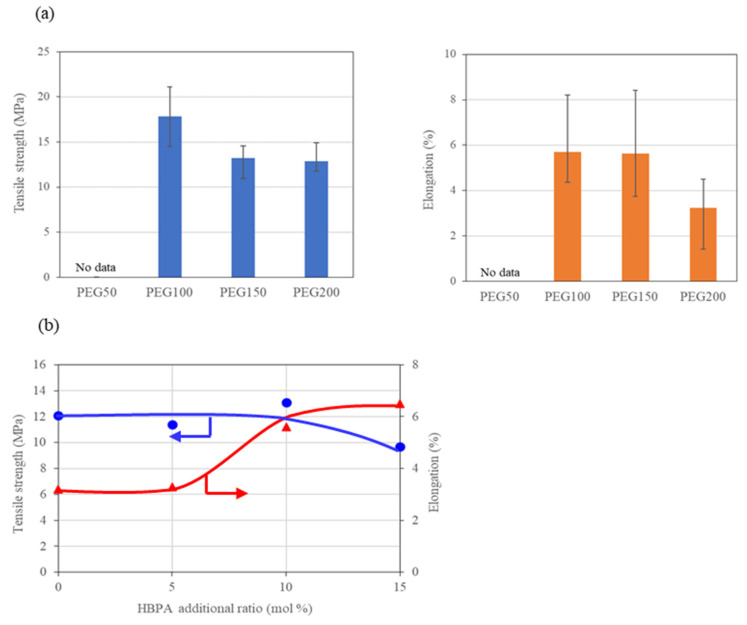
Tensile strength and elongation at breakage for the tensile tests of the epoxy monolith fabricated from (**a**) the TETRAD-C/BACM/PEG system and (**b**) the TETRAD-C/HBPA/BACM/PEG200 system. (●) Tensile strength and (▲) elongation.

Figure 6a shows the tensile strength and elongation at the point of breakage of the monolith sheets (thickness: 200–250 μm, N = 6). The figure includes no data for PEG50 because the epoxy monolith with the largest phase separation structure could not maintain its whole sample shape. Both the strength and elongation values for the monolith sheets become higher when PEGs with lower molecular weights are used. As shown in Figure 3, the smaller PEGs provide epoxy monoliths with larger pores. It is interesting that the presence of coarse pores exhibits favorable mechanical properties, such as high strength and high elongation. Figure 6b shows the effect of the addition of HBPA on the tensile strength and elongation. As shown in Figure 5, the pore diameter increases according to the amount of HBPA added to the curing system. The tensile strength is constant, regardless of the HBPA amount. With the addition of 10 mol% of HBPA, the elongation drastically increased. The test pieces that included more than 20 mol% of HBPA were too brittle, and thus difficult to use for determining the mechanical properties. In the systems that were controlled by the molecular weights of the PEGs and the amounts of HBPA added, the relationship between the pore sizes and the tensile strength was inconsistent. This is due to that the network density of the cured epoxy also decreases with the addition of HBPA, resulting in a decrease in the tensile strength of the monoliths. In contrast, the elongation values increase when the pore size is larger for both series, a fact that demonstrates a positive agreement with the results previously reported [26].

**Figure 7:**
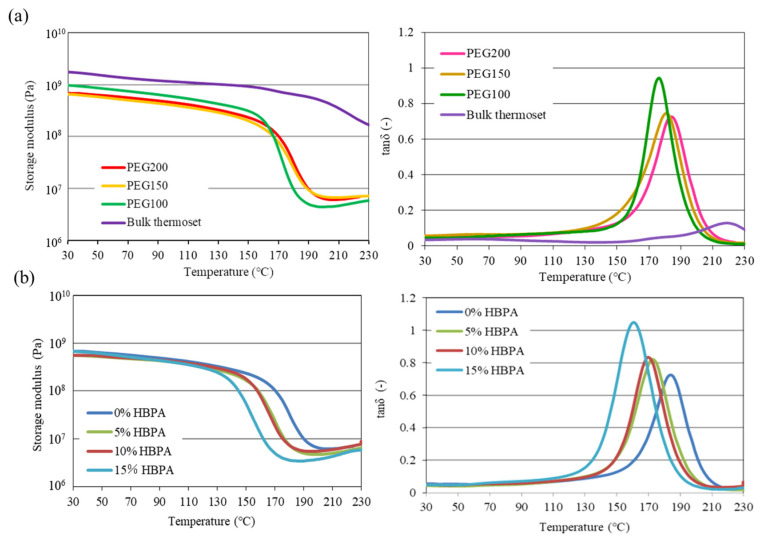
DMA curves for the epoxy monoliths and the bulk thermoset fabricated under various additive conditions. (**a**) Epoxy monoliths prepared in the presence of various PEGs and bulk thermoset prepared in the absence of PEG, and (**b**) epoxy monoliths prepared with the addition of various amounts of HBPA.

Figure 7 shows the results for the DMA measurement of the epoxy monoliths prepared under various additive conditions. The structural, mechanical, and physical properties of the monoliths and the bulk thermoset are summarized in Table 3. The epoxy bulk thermoset obtained using TETRAD-C and BACM without any porogenic solvent was originally a tough resin with a high elastic modulus (1.8 GPa), and which maintained evenness at high temperatures, since the Tg value of the bulk thermoset reached a value as high as 223 °C, as shown in Figure 7a and Table 3. In contrast, the epoxy monoliths prepared in the presence of PEGs exhibited an elastic modulus less than 1 GPa at room temperature, and the Tg values were as low as 175–187 °C. The Tg values slightly increased with an increase in the molecular weights of the PEGs. The softening characteristics of the epoxy monolith arise due to the plasticizing effect caused by the PEGs incorporated into the epoxy monolith skeletons during the fabrication process of the monolith, as was pointed out in our previous report [33]. Figure 7b shows the effect of the addition of HBPA on the DMA properties. The properties of the cured epoxy containing HBPA reflected the softening effects caused by the presence of bifunctional epoxy units, resulting in a slight decrease in the Tg value and an increase in the elongation at breakage. Thus, it has been demonstrated that it is possible to fabricate epoxy materials with the desired mechanical and thermal properties by the introduction of a monolith structure into hard epoxy resin.

### 3.4. Adhesive Property

**Figure 8:**
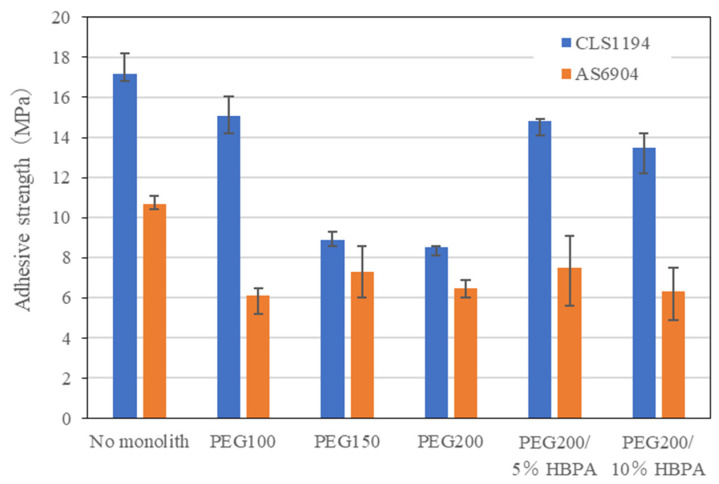
Comparison of adhesive strength for the lap-shear tensile test of SPCC using composite adhesive materials consisting of epoxy monolith sheets and commercial epoxy and acrylic adhesives.

Figure 8 shows the results for the adhesion test of SPCC using the monolith sheets fabricated in this study in combination with commercial adhesives. The test pieces used for the lap-shear tensile test were prepared using SPCC plates as adherends and the commercially available and most typical epoxy or acrylic adhesives in the presence of an epoxy monolith sheet (with a thickness of 80–120 μm) used as the adhesive layer (see Figure 2). As a result, the installation of an epoxy monolith sheet onto an adhesive layer resulted in a decrease in the lap-shear strength in both cases using the epoxy and acrylic adhesives (CLS1194 and AS6704). The adhesive strength of the epoxy adhesive varied depending on the kind of monolith sheets with different pore sizes. On the other hand, the constant strength value was obtained using the acrylic adhesive. This difference occurred due to the viscosity of the adhesive. The viscosities of CLS1194 and AS6704 are 4.0 Pa-s and 0.10 Pa-s, respectively. Thus, the penetration of the epoxy adhesive with high viscosity into the small pores of the monolith is difficult in cases involving the usage of PEG150 and PEG200. Moreover, the deterioration of adhesive strength is attributable the fact that the monolith layer was more likely to break in comparison with the higher storage modulus values of the adhesive. 

Thus, we have succeeded in the demonstration of a new type of bonding using epoxy and acrylic adhesives in combination with epoxy monolith sheets. Because there are great operational advantages for using a liquid adhesive as the state impregnated in the epoxy monolith sheet, the future challenge is to achieve greater adhesive strength by optimizing the physical properties of the monolith sheet. Thus, in the future, monolith adhesive structures will be applied, regardless of the type of adhesive.

## 4. Conclusions

We fabricated epoxy monoliths using TETRAD-C as the tetrafunctional epoxy and BACM as the amine curing agent in the presence of PEGs with different molecular weights used as porogenic solvents. It has been demonstrated that we can control the pore diameter and mechanical properties by utilizing the differences of the molecular structures and solubility parameters of porogenic solvents. This also affects the mechanical and physical properties of epoxy monoliths, such as the tensile strength, elongation, elastic modulus, and Tg values. For example, epoxy monoliths prepared in the presence of PEGs exhibited an elastic modulus less than 1 GPa at room temperature and the Tg values of 175–187 °C, while the epoxy bulk thermoset produced without any porogenic solvent showed a high elastic modulus of 1.8 GPa, which is maintained at high temperatures, and a high Tg of 223 °C. It has been revealed that adhesive structure consisting of an epoxy monolith sheet in combination with commercial epoxy and acrylic adhesives functions as a sheet-type adhesive. The test pieces for the lap-shear tensile test were prepared using SPCC plates as adherends and commercially available epoxy and acrylic adhesives in the presence of an epoxy monolith sheet. The installation of an epoxy monolith sheet onto an adhesive layer resulted in a slight decrease in the lap-shear strength, but a constant strength value was obtained using an acrylic adhesive. The deterioration of adhesive strength is attributable the fact that the monolith layer was more likely to break in comparison with the higher storage modulus values of the adhesive. In the case of the single-use of liquid adhesives, it is necessary to regulate application procedures and carefully control the thickness of the adhesives. In contrast, these troublesome and time-consuming problems could be avoided with the new adhesion method using the monolith sheets.

## Figures and Tables

**Table 1:** Solubility parameters of the materials used for monolith fabrication.

Category	Materials	Solubility Parameter ^a^ (−)
Epoxy	TETRAD-C	10.1
	HBPA	9.2
Amine curing agent	BACM	9.7
Porogenic agent	PEG50	14.1
	PEG100	12.6
	PEG150	12.1
	PEG200	11.6

^a^ Estimated using the Fedors method [38].

**Table 2:** Peak temperature and enthalpy for the thermal curing of the TETRAD-C/HBPA/BACM/PEG system determined by DSC.

HBPA Content (mol%)	PEG	Onset Temperature (°C)	Peak Temperature (°C)
0	PEG50	69	112
0	PEG100	74	120
0	PEG150	79	133
0	PEG200	86	139
5	PEG200	72	138
10	PEG200	72	138
15	PEG200	71	138
20	PEG200	70	136
30	PEG200	68	136

**Table 3:** Structural, mechanical, and physical properties of monolith sheets prepared in the presence or absence of PEGs and HBPA.

HBPA (mol%)	PEG	Mean Pore Diameter (μm)	Tensile Test		DMA	
			Strength (MPa)	Elongation (%)	*T*_g_ (°C)	Storage Modulus at 25 °C (GPa)
0	PEG50	3.80	-	-	-	-
0	PEG100	1.91	17.9	5.8	175	1.01
0	PEG150	0.63	13.3	5.6	177	0.66
0	PEG200	0.28	12.5	3.2	187	0.68
5	PEG200	0.87	11.4	3.3	172	0.56
10	PEG200	1.21	13.2	5.6	170	0.56
15	PEG200	3.15	9.5	6.5	161	0.59
0	-	-	-	-	223	1.81

## Data Availability

The data presented in this study will be available on request.

## Supplementary Materials

The following supporting information can be downloaded at: https://www.mdpi.com/article/10.3390/molecules29092059/s1, Figure S1: The procedure for preparing an epoxy monolith sheet; Figure S2: DSC traces for thermal curing of the epoxy systems; and Figure S3: Photographs of the fracture surfaces of the test pieces after the tensile test in Figure 8.
