# Supplementary material for: Control of Pore Sizes in Epoxy Monoliths and Applications as Sheet-Type Adhesives in Combination with Conventional Epoxy and Acrylic Adhesives
Source: Molecules. 2024 Apr 29;29(9):2059. doi: 10.3390/molecules29092059 (PMC11085113; doi:10.3390/molecules29092059)
Supplement: Supplementary file 1 [file molecules-29-02059-s001.zip › molecules-2956616-supplementary.pdf]

Supplementary materials for Molecules

Control of Pore Sizes in Epoxy Monoliths and Applications as Sheet-type Adhesives in Combination with Conventional Epoxy and Acrylic Adhesives

Yoshiyuki Kamo and Akikazu Matsumoto

Contents

Figure S1. The procedure for preparing an epoxy monolith sheet.

Figure S2. DSC traces for thermal curing of the epoxy systems.

Figure S3. Photographs of the fracture surfaces of the test pieces after the tensile test in Figure 8.

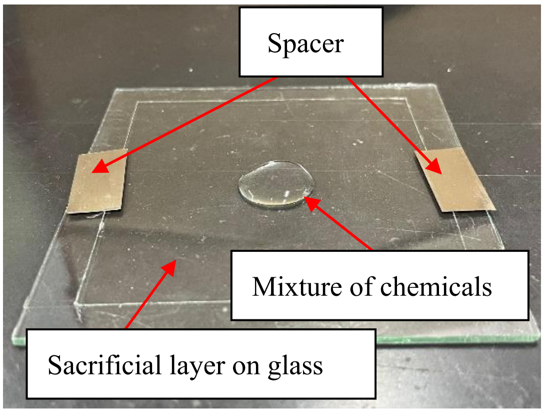

Step 1. A mixture of epoxy, amine, porogenic solvent was spread on a glass plate precoated with a sacrificial layer of PVA.

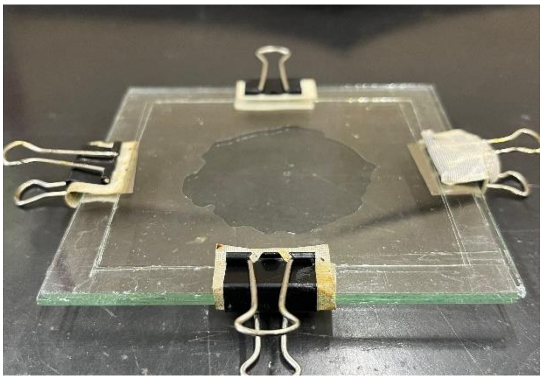

Step 2. The mixture was sandwiched with another glass plate while maintaining the desired thickness using spacers and then cured at 130°C for 30 minutes.

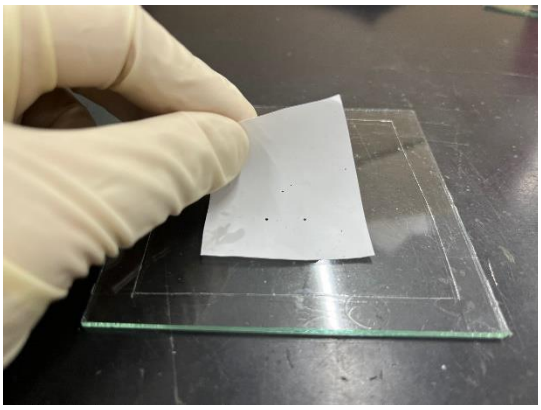

Step 3. The monolithic sheet was peeled off the glass after curing. The sheet was washed in ion-exchanged water using ultrasonics for 15 min.

Figure S1. The procedure for preparing an epoxy monolith sheet.

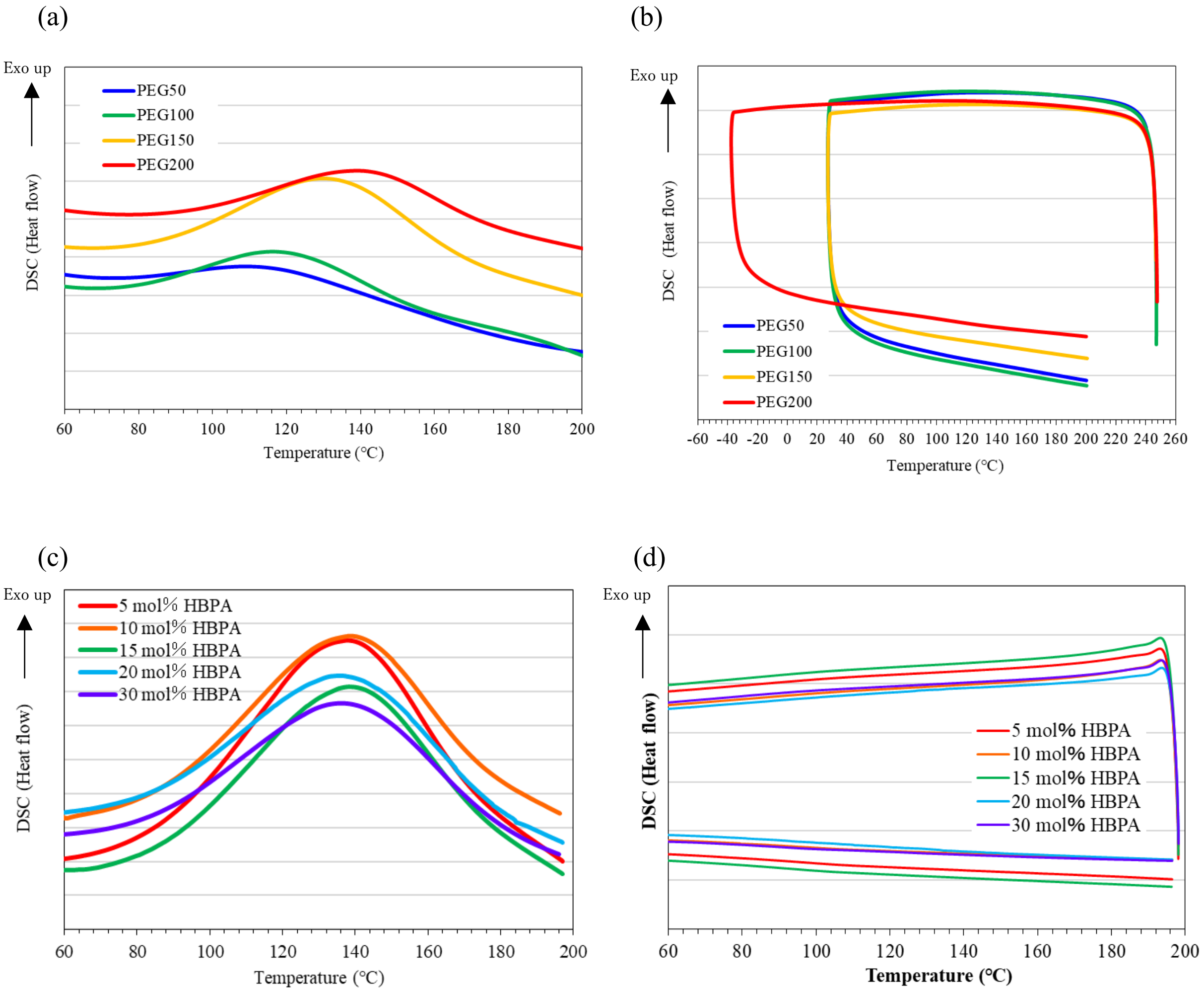

Figure S2. DSC traces for thermal curing of the epoxy systems.

(a) Effect of the type of PEGs for the TETRAD-C/BACM/PEG systems and (b) second DSC curve for the same systems. (Initially, we conducted a detailed investigation on PEG200 from -50 to 250°C.)

(c) Effect of the HBPA content for the TETRAD-C/HBPA/BACM/PEG200 system (d) second DSC curve for the same systems.

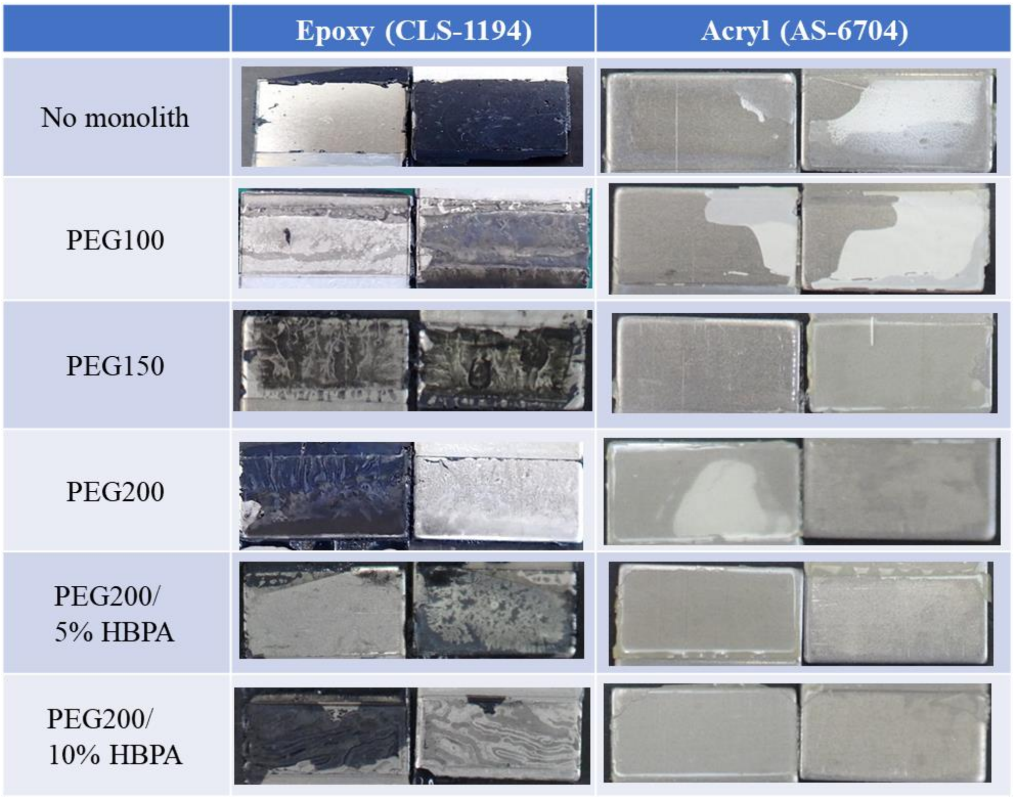

Figure S3. Photographs of the fracture surfaces of the test pieces after the tensile test in Figure 8.
